# Electronic Effect on the Molecular Motion of Aromatic Amides: Combined Studies Using VT-NMR and Quantum Calculations

**DOI:** 10.3390/molecules23092294

**Published:** 2018-09-08

**Authors:** Sungsoo Kim, Jungyu Kim, Jieun Kim, Daeun Won, Suk-Kyu Chang, Wansik Cha, Keunhong Jeong, Sangdoo Ahn, Kyungwon Kwak

**Affiliations:** 1 Department of Chemistry, Chung-Ang University, Seoul 156-756, Korea; hesperus86@gmail.com (S.K.); wldms01477@gmail.com (J.K.); panda881020@gmail.com (D.W.); skchang@cau.ca.kr (S.-K.C.); 2 Center for Molecular Spectroscopy and Dynamics, Institute for Basic Science (IBS), Seoul 02841, Korea; kimjoongyu@naver.com; 3 Department of Chemistry, Korea University, Seoul 02841, Korea; 4 Nuclear Chemistry Research Division, Korea Atomic Energy Research Institute, Daejeon 34057, Korea; wscha@kaeri.re.kr; 5 Department of Chemistry, Korea Military Academy, Seoul 01805, Korea

**Keywords:** rotational barrier energy, amide bond, nuclear magnetic resonance, kinetic, density functional theory

## Abstract

Rotational barrier energy studies to date have focused on the amide bond of aromatic compounds from a kinetic perspective using quantum calculations and nuclear magnetic resonance (NMR). These studies provide valuable information, not only regarding the basic conformational properties of amide bonds but also the molecular gear system, which has recently gained interest. Thus, we investigate the precise motion of the amide bonds of two aromatic compounds using an experimental rotational barrier energy estimation by NMR experiments and a theoretical evaluation of the density functional theory calculation. The theoretical potential energy surface scan method combined with the quadratic synchronous transit 3 method and consideration of additional functional group rotation with optimization and frequency calculations support the results of the variable temperature ^1^H NMR, with deviations of less than 1 kcal/mol. This detailed experimental and theoretical research strongly supports molecular gear motion in the aromatic amide system, and the difference in kinetic energy indicates that the electronic effect from the aromatic structure has a key role in conformational movements at different temperatures. Our study provides an enhanced basis for future amide structural dynamics research.

## 1. Introduction

The amide bond, a basic unit of proteins, has unique steric and energetic characteristics [1,2]. Therefore, studying the dynamics of these bonds is important to understand protein dynamics. Conformational studies of aromatic amide bonds, in particular, have been extensively carried out owing to their potential applications in fields such as asymmetric synthesis, molecular gear systems, single-molecule motors, and single-molecule devices [3].

An aromatic amide has two rotational motions [4] around the central carbonyl group: the rotation around aryl-CO and the rotation around the C-N bond in the amide unit. Therefore, the basic structural change of an aromatic amide involves three bond rotational processes [4,5] that compose the interconversions of the conformers. The two independent rotations of the C-N bond or aryl-CO bond interconvert two conformers (exo and endo forms), and the concerted rotation of these bonds represents the molecular gear. Both rotations and their three rotational processes [6,7] have received intense interest because rotational dynamics can be applied to single molecule dynamic studies and experimental data on rotational dynamics can be achieved with variable temperature nuclear magnetic resonance (VT-NMR) spectroscopy [8,9,10] in order to investigate the Gibbs free energy of the concerted C-N/aryl-CO bond rotation and/or independent aryl-CO and C-N bond rotation around the carbonyl group on the aromatic amide. These rotations can be controlled through chemical modification of the structure or simple temperature variation [11].

The internal rotation [12] around the C-N bond in the amides provides information regarding the conformational characteristics of the backbone of peptides and proteins. There have been many reports concerning determination of the steric effect involving rotational dynamics [5,6], although few of these have discussed electronic effects owing to the difficulty of separately measuring the steric and electronic effects. The other rotational dynamic of aromatic amides, aryl-CO, has been successfully explained in the case of the C-C single bond rotational process in *N*-methylbenzamide [7]. However, the electron-rich aromatic ring in molecular gear studies is usually designed and connected to the *N* of amide groups. Thus, the aryl-CO bond is correlated to the C-N bond in many experimental studies [11,13,14]. It is therefore important to study the electronic effects of aromatic amides, including aryl-CO and C-N bonds, using both theoretical and experimental methods, which will clearly explain the molecular motion. Herein, we use quantum calculations and VT-NMR to investigate the rotational process of aryl-CO and C-N bonds in specifically designed *N*,*N*-diethylamide derivatives.

## 2. Results

### 2.1. Interpretation of VT-NMR: Coalescence

#### Characterization of *N*,*N*-diethylamide Derivatives (1), (2)

**Figure 1:**
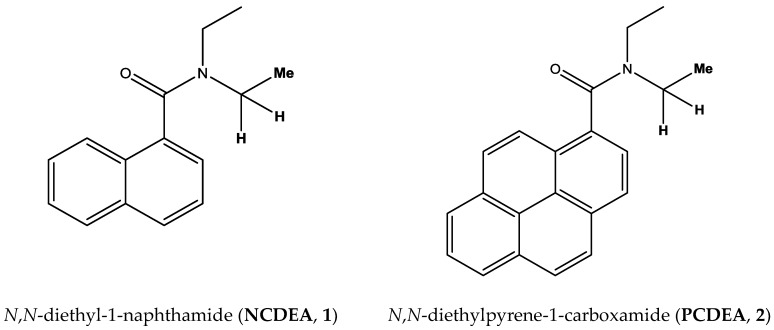
Structures of *N*,*N*-diethylamide derivatives.

*N*,*N*-diethylamide derivatives (1), (2) were synthesized to measure the rotational barrier of the aryl-CO and C-N bonds (Figure 1). Before we evaluated the rotational barrier using ^1^H VT-NMR, the H and Me peaks from two ethyl groups attached to N were assigned to understand which rotations were occurring. The peaks of chemical shifts in *N*,*N*-diethylpyrene-1-carboxamide (PCDEA) have a similar trend to those in *N*,*N*-diethyl-1-naphthamide (NCDEA) at 25 °C. As shown in Table 1, the ^1^H NMR spectrum in NCDEA has two H chemical shift peaks (δ 3.51 ppm, δ 3.82 ppm), as does the ^1^H NMR spectrum in PCDEA (δ 3.64 ppm, δ 3.89 ppm). These two peaks occur because two H atoms are located differently with respect to each aromatic group, resulting in a more de-shielded proton signal when the position is closer to the aromatic group. Similarly, Me peaks are better shielded than H peaks in PCDEA (δ 1.32 ppm, δ 1.77 ppm) and in NCDEA (δ 0.98 ppm, δ 1.34 ppm).

**Figure 2:**
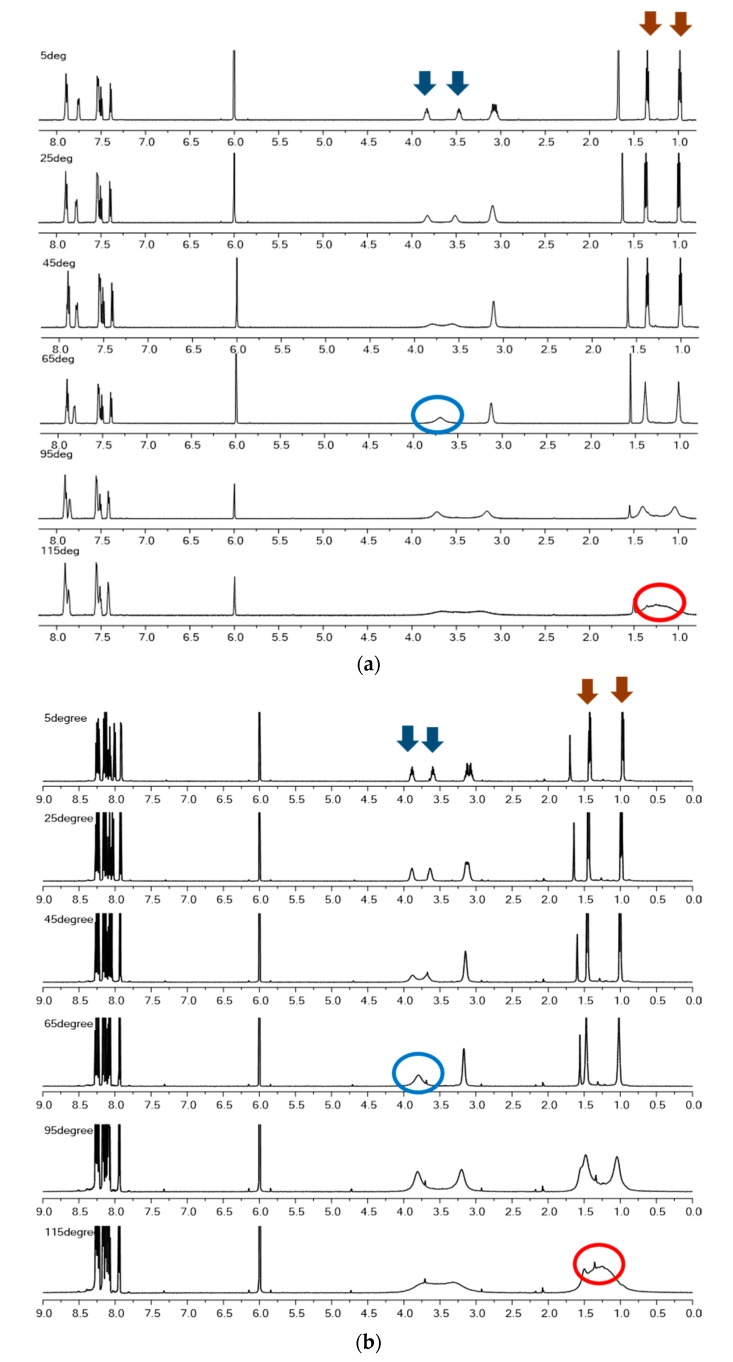

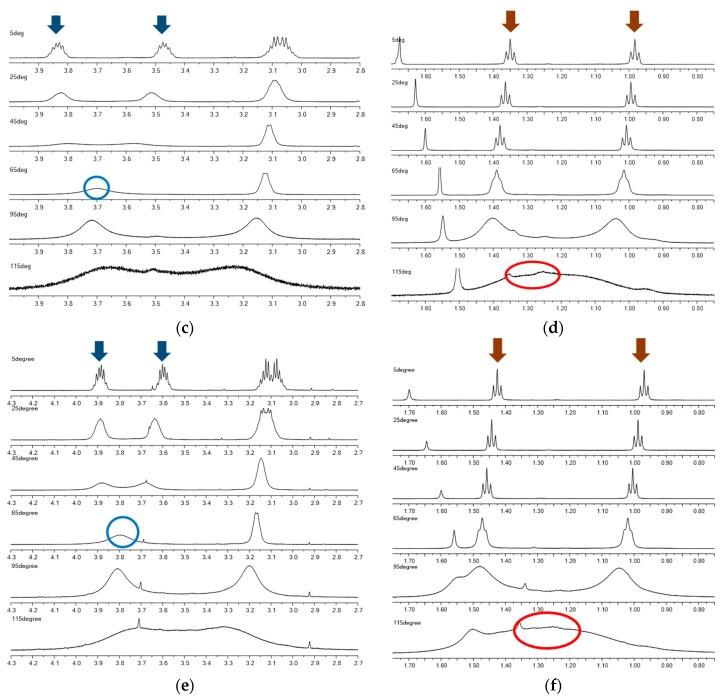
^1^H VT-NMR experiments on NCDEA and PCDEA. Arrows indicate each H and Me signal in each structure, and circles indicate the merged signals after the temperature increase. (**a**) Whole spectrum from NCDEA. Arrows indicate split H and Me signal and circles indicate the merged signal after the temperature increase. (**b**) Whole spectrum from PCDEA. Arrows indicate split H and Me signal and circles indicate the merged signal after the temperature increase. (**c**) H-H merges as temperature increases (NCDEA). (**d**) Me-Me merges as temperature increases (NCDEA). (**e**) H-H merges as temperature increases (PCDEA). (**f**) Me-Me merges as temperature increases (PCDEA).

Line shape analysis of the ^1^H VT-NMR experiment was applied to evaluate the relative Gibbs free energy difference and the enthalpy difference for aryl-CO and C-N rotation. Figure 2 shows that the experimental values are in good agreement with the theoretical analysis. The following (Eyring equation) estimates ${\Delta G}^{‡}$ in ^1^H VT-NMR using the coalescence temperature from the two-site exchange [9,15].

(1) $${\Delta G}^{‡}=0.01914\times \mathrm{Tc}\times \left[10.319+\log_{10}\left(\frac{Tc}{k}\right)\right],\;\left(k=\frac{\pi \Delta \upsilon }{\sqrt{2}}\right)$$

In the experiment, the split NMR signals are from two ground states. If their exchange rate constant by rotation is greater than their frequency separation, the peaks are merged into one. Therefore, if two peaks are merged into one peak, the rotational barriers of aryl-CO and C-N and the rate constant are detected by the coalescence temperature. The rate constant (*k*) has a fast exchange for aryl-CO and C-N rotations.

As the temperature increases, the merging of H-H into one relatively sharp peak corresponds to aryl-CO, and merging of Me-Me into one broader peak represents concerted aryl-CO/C-N rotation, which is well supported by a previous extensive theoretical study [16].

NCDEA has two aryl-CO and C-N bond rotations. Two different characteristics are explained by the peak coalescence of the ^1^H VT-NMR experiment as the temperature increases. The spectra show that aryl-CO rotation represents the broadening signals of H-H peaks from δ 3.60 to δ 3.80 ppm (Figure 2b). As the temperature increases, the signals of Me-Me from δ 1.0 to δ 1.4 ppm are assigned to the C-N rotation (Figure 2c). At 65 °C, it is clear that two peaks coalesce in the aryl-CO bond rotation, as shown in Figure 2. On the other hand, the coalescence temperature of the C-N rotation is higher than that of the aryl-CO rotation (Table 2).

PCDEA exhibits a similar spectral pattern in the ^1^H VT-NMR experiment. At the coalescence temperature, the chemical shifts representing aryl-CO bond rotation range from δ 3.70 to δ 3.90 ppm (Figure 2e), which is similar to the case of NCDEA. C-N bond rotation is shown from δ 1.00 to δ 1.50 ppm when coalescence begins, and again, is very similar to that of NCDEA (Figure 2f).

A two-dimensional exchange spectroscopy (EXSY) experiment was performed to confirm the rotational dynamics at high temperatures (Figure S1). The mixing time was 200 ms, and ω_o_τ_c_ ≪ 1 (ω_o_ = spectrometer frequency in radians; τ_c_ = rotational correlation time) was satisfied; thus, the nuclear overhauser effect (NOE) of H in molecules is not expected to exist [17]. Therefore, the cross-peaks shown in Figure S1 can only be expressed by chemical exchange mechanisms. The 2D EXSY spectrum shows how each bond rotation progresses at three increasing temperatures.

### 2.2. Theoretical Calculations

#### 2.2.1. One-Dimensional Potential Energy Surface Scan for Aryl-CO and C-N Rotation

Many potential energy surface (PES) studies have been reported, detailing experimental results that are consistent with the computational analysis of the molecules of interest. Likewise, we investigated the energy based on the geometric optimization of scan coordinates, changing the dihedral angles from −170° to 200° for the C-N bond and aryl-CO bond, respectively, in *N*,*N*-diethylamide derivatives (1), (2). In the ^1^H VT-NMR results, two types of bond rotation characteristics were detected on aryl-CO and C-N bonds, either simultaneously or separately. NCDEA and PCDEA have discrete values of chemical shifts, as explained previously. The scan coordinates of those molecules on C-N and aryl-CO bonds were calculated separately.

When the aryl-CO bond rotates, the diethyl group attached to the N is directly confronted with the steric effect from the naphthalene (1) and pyrene (2) rings, which increases the optimized energy representing repulsion (Figures S2a and S3a). On the other hand, when concerted aryl-CO and C-N bond rotation occurs, all the diethylamide derivatives manifest a similar tendency for molecular orientations and elucidate the concurrent rotations of aryl-CO and C-N bonds; thus, there are two similar transition states (Figures S2b and S3b).

We determined that the PES of the C-N bond rotation of NCDEA and PCDEA indicates that the signals of peak coalescence in VT-NMR show similar chemical shifts. This suggests that the PES of aryl-CO and C-N bond rotations have two ground states, which are enantiomers, and two transition states, indicating that they have two rotational barriers. The Gibbs free energy between the transition state and ground state approximately demonstrates the experimental NMR analysis (Table S1). However, the true transition states would enable a more accurate theoretical description of the actual Gibbs free energy difference.

#### 2.2.2. Calculated Result for QST3 and Changing Dihedral Angles of Di-ethyl Groups

##### Scheme for Interconversion in *N*,*N*-Diethylamide Derivatives (1), (2)

**Figure 3:**
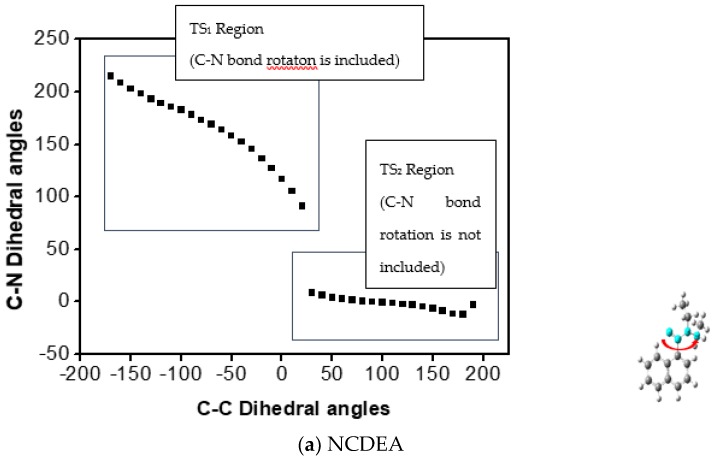

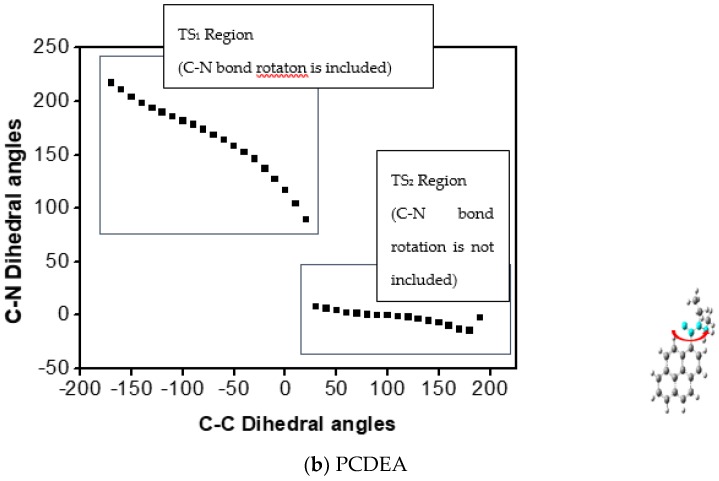
Dihedral angle of C-N bond (highlighted) (Y axis) versus dihedral angle of Aryl-CO bond (Arrowed) (X axis) in (**a**) NCDEA and (**b**) PCDEA.

**Figure 4:**
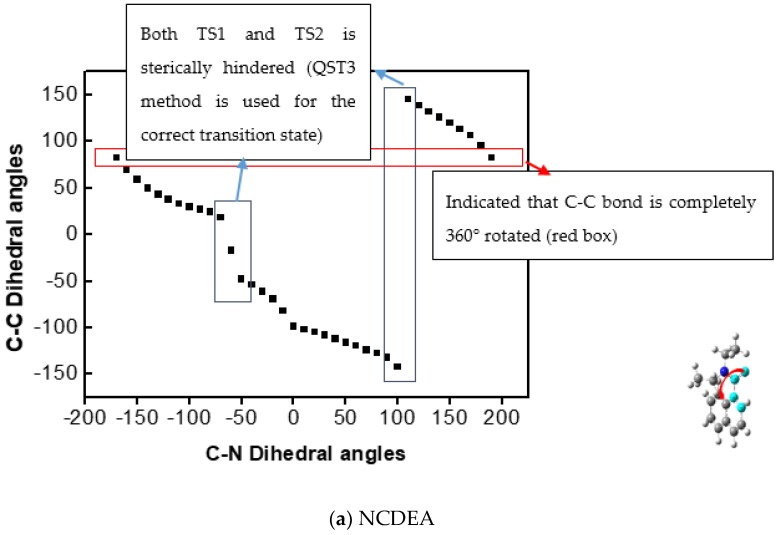

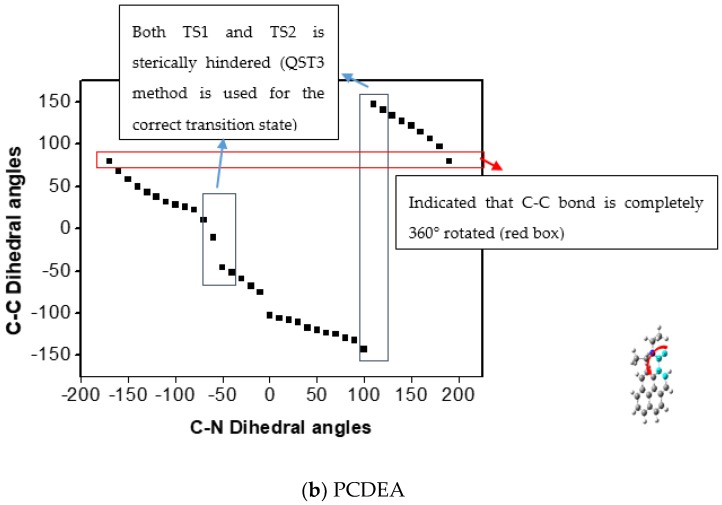
Dihedral angle of Aryl-CO bond (Highlighted) (Y axis) versus dihedral angle of C-N bond (Arrowed) (X axis) in (**a**) NCDEA and (**b**) PCDEA.

Before the transition states were obtained through quadratic synchronous transit 3 (QST3) [18], we obtained the change in dihedral angle of the aryl-CO bond as the dihedral angle of the C-N bond increased, or vice versa, by 1D PES analysis. As indicated in Figure 3 and Figure 4, whereas aryl-CO bond rotation is independent of C-N rotation, C-N bond rotation has an influence on aryl-CO bond rotation in that it experiences concerted rotation with the aryl-CO bond, consistent with previous NMR and calculation results.

##### Transition States Optimized through the QST3 Method

The two ground states are two types of structure, i.e., enantiomers, that exhibit the same energy, which is supported by the following energy calculation. Diethyl conformations of the two ground states of *N*,*N*-diethylamide derivatives are in opposite directions if the benzene ring group is considered a mirror plane (Figure S4).

For aryl-CO bond rotation of NCDEA and PCDEA, the barrier of the first transition state (1TS) is different from that of the second transition state (2TS) in that the rotational motion of 1TS in the aryl-CO bond contains C-N bond rotation when the PES scan is performed. This originates from the steric hindrance between naphthalene/pyrene rings and diethyl groups, resulting in C-N bond rotation. Thus, we do not have to consider the Gibbs free energy of 1TS (hence QST3-1TS of the aryl-CO bond is not included in Figure S4 or Table S2).

The scan coordinates do not confirm whether the two transition states found in this way are structurally true transition states, which are required for an adequate Gibbs energy difference, even though they have one negative imaginary frequency. Other optimized processes (QST3) for finding true transition states are required to subsequently characterize the structure and energy of each state (Figures S2 and S3); these optimized structures also have imaginary frequencies in which the mode corresponds to rotation of the aryl-CO bond and the concerted aryl-CO/C-N bond, respectively (Table S2). The structures of two GSs and one or two TSs for *N*,*N*-diethylamide derivatives (1), (2) are expressed as dihedral angles (Schemes S2 and S3) in order to more easily understand the energy difference (Figure S4). Furthermore, the study of intrinsic reaction coordinates analysis is added for confirming the transition state.

##### Dihedral Angle Change in the Diethyl Conformation of Transition States of the Aryl-CO Bond in NCDEA and PCDEA

Changing the dihedral angles of the diethyl moiety of transition states is necessary for explaining the routes to rotational barriers. As we described earlier, since the 1TS of aryl-CO bond rotation includes C-N rotation, and the increase of activation energy and energy of 1TS in aryl-CO/C-N bond rotation is greater than that of 2TS after the QST3 calculation, we only consider the 2TS of the aryl-CO bond and aryl-CO/C-N bond rotation and make the dihedral angle change for the proper theoretical value consistent with the experimental value. Results of calculations with various dihedral angles are described in detail, and these energy values are very close to the experimental data (Tables S3–S5).

In particular, for the energy value of aryl-CO bond rotation in NCDEA, the theoretical Gibbs free energy value can range from 14.24 to 15.19 kcal∙mol^−1^, a range of less than 1 kcal∙mol^−1^, which is highly consistent with the NMR experimental results; four of the di-ethyl rotation modes, however, do not correspond to aryl-CO bond rotation with more than an imaginary frequency (Table S3).

PCDEA shows a similar tendency in aryl-CO and C-N bond rotation to that of NCDEA. It is clear that the value estimated by di-ethyl rotation is similar to the experimental value. The importance of applying the dihedral angle change is thus confirmed; the value of the Gibbs free energy for QST3 optimization is 13.52 kcal∙mol^−1^ and can be increased by di-ethyl rotation to 14.37 and 15.74 kcal∙mol^−1^, values that are only approximately 1 kcal lower than or the same as, respectively, the experimental values (Table S4).

###### Transition State of the 2D PES

**Figure 5:**
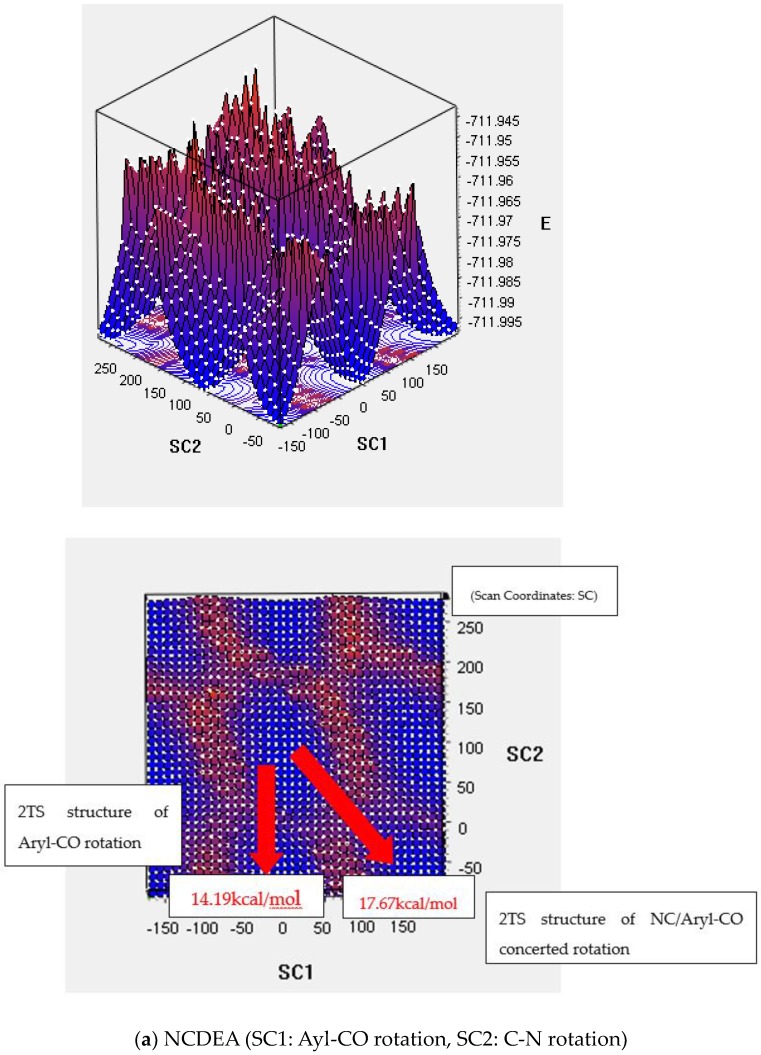

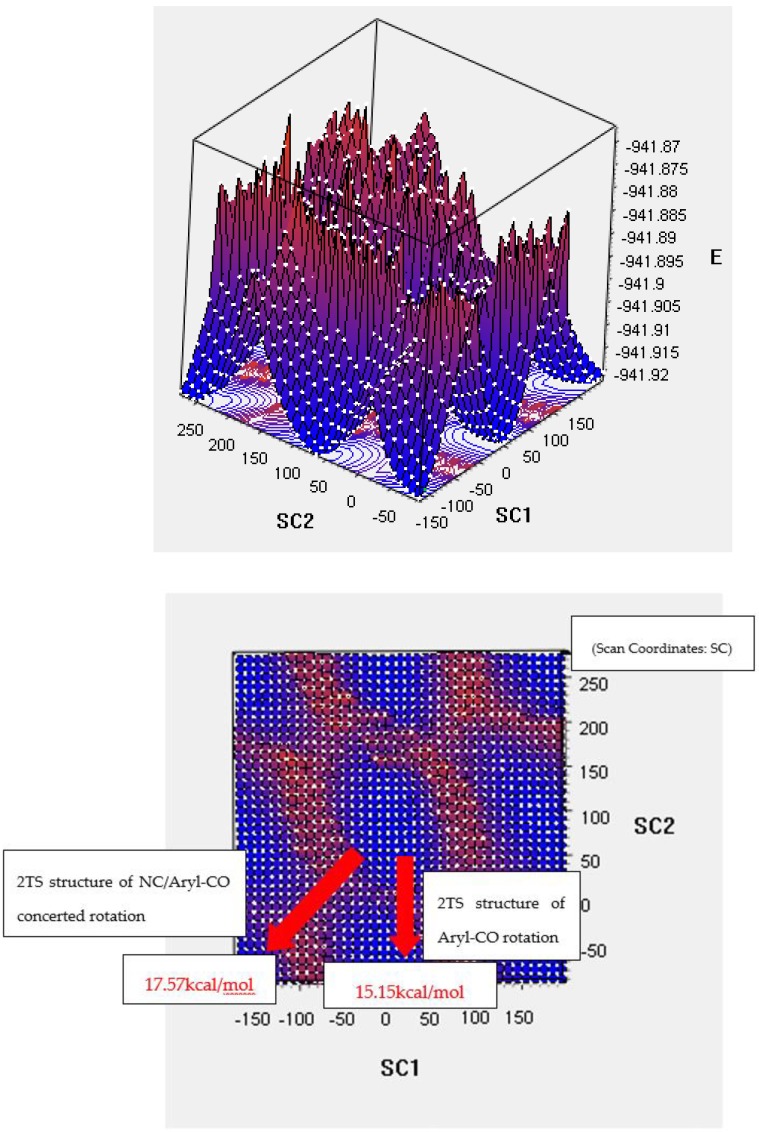
2D-Potential Energy Surface on Aryl-C = O Dihedral angle and C-N Dihedral angle change of NCDEA (**a**) and PCDEA (**b**).

As indicated in the 2D PES scan with frequency calculation in Figure 5, we also investigated independent aryl-CO and C-N dihedral angles for concerted aryl-CO/C-N bond rotation. As shown in Figure 4, as the C-N dihedral angle (X axis) decreases, the aryl-CO (Y axis) increases so that NCDEA and PCDEA have different Gibbs free energies for aryl-CO bond rotation (NCDEA ${\Delta G}^{‡}$: 14.19 kcal and PCDEA ${\Delta G}^{‡}$: 15.15 kcal) and C-N bond rotation (NCDEA ${\Delta G}^{‡}$: 17.67 kcal and PCDEA ${\Delta G}^{‡}$: 17.57 kcal). This means that C-N bond rotation affects the aryl-CO bond rotation, as it is a molecular gear model [3]. On the other hand, aryl-CO bond rotation does not affect C-N bond rotation; thus, it rotates independently. This means that the ethyl group connected to the tertiary amine could be sterically hindered by aromatic group when it rotates around. Those results robustly support our previous theoretical and experimental data. Thus, we confirmed via both of Gibbs free energy values (Table 3) and the transition state structures, that the transition state structure of C-N bond rotation is consistent with the structure of the concerted C-N/aryl-CO bond in 2D PES (Tables S6–S10).

###### Quantum Theory of Atoms in Molecules (QTAIM) Study

The information from the QTAIM study qualitatively supports all the results of this study. The electron density of the C-N/aryl-CO bond in the transition state is generally greater than that of the aryl-CO bond in each structure, demonstrating that the Gibbs free energy of C-N/aryl-CO rotation is greater than that of aryl-CO. Furthermore, the electronic effect from different aryl groups shows the different electron populations on the rotating bonds in the transition state, which reveal the Gibbs free energy difference between NCDEA and PCDEA (Table S11).

## 3. Discussion

Rotational barrier energy studies of amide bonds of aromatic compounds from a kinetic perspective provide valuable information regarding the basic conformational properties of amide bonds and their molecular gear system, which have been of recent scientific interest. To that end, we investigated rotational barrier energy using two methods to determine the precise motion and theoretical quantum chemical effects using an NMR experiment and density functional theory [19,20]. The theoretical PES scan method, with optimization and frequency calculations suggested by the theoretical study, supports the effect of ^1^H VT-NMR, which assumes that if the bond rotation is fast enough to break out the double bond characteristic of interest, the two peaks are merged and cannot be distinguished from chemical shifts. Thus, we can predict and evaluate the energy barrier using the Eyring equation [9,15].

By quantum chemical methods, the PES can be predicted correctly for rotation in a simple system such as ethane (~3 kcal∙mol^−1^) [21]. The PES for diethylamide derivatives, however, has a limitation in explaining the structure of transition states, such that the energy barrier might be miscalculated. This means that the transition states must be re-calculated using QST3 optimization [18], followed by frequency calculation, which shows us that each state has an imaginary frequency. This clearly shows that the transition states assumed to be true demonstrate the rotational energy barriers in the NMR experiment.

The final step, di-ethyl rotations, is required to determine whether each transition states’ energy can be varied in the range of several kcals, revealing that the computational result from this step is consistent with energy values from the NMR experiment (Table 3). To further investigate the rotational dynamics, we performed QTAIM studies [22]. The analysis qualitatively supported the Gibbs free energy difference and electronic effect from the different molecular structures. This detailed experimental and theoretical research strongly supports the molecular gear motion for the aromatic amide system and the difference in kinetic energy, provided that the electronic effect from the aromatic structure plays a key part in the conformational movement at different temperatures. Our study provides a basis for future research on amide structural dynamics.

## 4. Materials and Methods 

### 4.1. Sample Preparation

We added oxalyl chloride to a mixture of pyrene-1-carboxylic acid and 1-naphthoic acid in methylene chloride and added dimethyl formamide after approximately 1–2 drops [23] (Scheme S1). The mixture was stirred at room temperature to maintain the reaction for approximately 4 h. Once the reaction was completed, the methylene chloride was evaporated, and 1 h of vacuum was performed. The dried yellow powder was then dissolved in methylene chloride. We added this mixture to a solution of diethylamine and methylene chloride by a dropwise method. This step was followed by extraction with D.W. three times. After sample preparation, we confirmed the product by thin layer chromatography and determined molecular structures with NMR, the results of which were used for data processing.

### 4.2. Experimental Methods

^1^H NMR spectra were obtained using a Varian VNS 600 spectrometer with a temperature control system. 1,1,2,2-tetrachloroethane-d_2_ purchased from Euriso-Top was used as the solvent for *N*,*N*-diethylamide derivatives. Each sample was injected into a 5-mm o.d. NMR tube to be analyzed. The chemical shifts of the spectra were obtained using the solvent peak as a reference.

In the VT-NMR experiment, prior to detection, the samples were left long enough for all samples to be in equilibrium at the designated temperature, so as to increase the reproducibility and accuracy of the results. The experiments were performed in the range of 273–388 K, considering the boiling point of the solvent and the limiting temperature of proton detection. The spectra were typically obtained at intervals of 10°. The interval was 2° beginning with the coalescence temperature. The possible error range was ±0.5 K.

Two-dimensional exchange spectroscopy (EXSY) [24] was performed at different temperatures for the two *N*,*N*-diethylamide derivatives (Figure 1) using a normal nuclear Overhauser effect spectroscopy (NOESY) pulse sequence [25].

### 4.3. Computational Methodss

Calculations were performed at the B3LYP/6-311G(d) level by means of the Gaussian 09 software package (Gaussian, Inc., Wallingford, CT, USA) [26]. To find the Gibbs free energy difference between the ground state and transition state, we used density functional theory (DFT) [19,20,27] at the B3LYP/6-311G(d) level [28] with the PCM model [29] (dielectric constant ε = 8.2 of 1,1,2,2-tetrachloroethane). Gibbs free energy difference was calculated under standard conditions (a temperature of 298.15K and a pressure of 1 atm).

#### 4.3.1. 1D PES Method

First, the calculations in redundant coordinates were performed at 38 steps of every 10° increment of the aryl-CO bond and C-N bond in NCDEA and PCDEA. Harmonic vibrational frequencies were calculated for all stationary points. For each optimized state, the frequency analysis showed the absence of imaginary frequencies, whereas each transition state showed a single imaginary frequency [30]. Visual inspection of the corresponding normal mode was used to confirm that the correct transition state had been found.

#### 4.3.2. QST3 Method and Changing Dihedral Angles of Diethyl Groups Method

After 1D PES calculations, two assumed transition states of each aryl-CO and C-N bond rotation were re-optimized in QST3 algorithms [18] to confirm transition states. For this calculation, two lowest energy states and an estimated transition state were required. After using the QST3 method, one transition state was calculated. Frequency calculations were also performed to find the imaginary frequency corresponding to bond rotations, meaning that one transition state of those bond rotations could be found.

Diethyl rotations result in a different Gibbs energy barrier; therefore, before we optimized the confirmed transition states for the aryl-CO bond and C-N bond in NCDEA and PCDEA, we prepared for the conformers, of which all the atoms except ethyl were fixed, and the dihedral angles (α, β) of C (one ethyl)-*N*-C (the other ethyl)-C (the other ethyl) were −60°, 60°, and 180°, respectively (Scheme S2). Thus, each conformer had nine different combinations (Scheme S2) and was optimized to have stable energy states. Frequency calculations were employed in these nine conformers to find the transition states of the aryl-CO and N-C bonds for selecting the optimum Gibbs energy barrier. We also performed intrinsic reaction coordinates of two transition states from QST3 algorithms and the nine conformers from 2nd transition states in aryl-CO and C-N bond rotations to find actual transition states including one imaginary frequency.

#### 4.3.3. 2D PES Method

The activation energy of aryl-CO and C-N bond rotation can be specifically determined by independently calculating aryl-CO as the X axis and C-N bond as the Y axis. 2D PESs were calculated for 38 steps of every 10° increment of aryl-CO and C-N bond rotations. Frequency calculations were performed in an estimated transition state in both NCDEA and PCDEA.

#### 4.3.4. QTAIM Study

In order to estimate the electron density on the rotating bonds (aryl-CO and C-N), we performed a QTAIM study using the Multiwfn program [22]. The electron density was calculated at the bond critical point (CP(3,−1)).

## Figures and Tables

**Table 1:** Experimental ^1^H NMR for *N*,*N*-diethylamide derivatives (aliphatic part only).

Compound	Signal	Chemical Shifts (δ)/ppm
NCDEA	H	3.51/3.82
NCDEA	Me	0.98/1.34
PCDEA	H	3.64/3.89
PCDEA	Me	1.32/1.77

**Table 2:** Bond rotation in *N*,*N*-diethylamide derivatives measured by ^1^H VT-NMR.

Compound	Coalescing Signals	Δυ/Hz	T_c_/°C	k/s^−1^	${\Delta G}^{‡}/\mathrm{kcal}\cdot {\mathrm{mol}}^{-1}$
NCDEA	H-H	175.43	60	602	15.40
NCDEA	Me-Me	273.93	115	389	18.00
PCDEA	H-H	219.62	57	478	15.62
PCDEA	Me-Me	220.28	115	478	17.65

**Table 3:** Comparison of the relative experimental Gibbs free energy differences to computational results.

		Variable Temperature ^1^H NMR	Theoretical Calculation (QST3//Di-ethyl Rotation *)
Compound	Bond	${\Delta G}^{‡}/\mathrm{kcal}\cdot {\mathrm{mol}}^{-1}$	${\Delta G}^{‡}/\mathrm{kcal}\cdot {\mathrm{mol}}^{-1}$
NCDEA	C-N/aryl-CO	18.00	1TS: 20.61 ** 2TS: 17.13//16.09~20.04 2D TS: 17.67
	aryl-CO	15.40	1TS: C-N bond rotation is included 2TS: 14.24//14.24~15.19 2D TS: 14.19
PCDEA	C-N/aryl-CO	17.65	1TS: 19.94 ** 2TS: 17.04//15.97~19.92 2D TS: 17.57
	aryl-CO	15.62	1TS: C-N bond rotation is included 2TS: 13.52//13.38~15.74 2D TS: 15.15

Di-ethyl rotation * represents energy values between minimum and maximum. ** 1TS showed higher Gibbs free energy than 2TS in QST3, which was not considered for di-ethyl rotation calculation.

## Supplementary Materials

The following are available online. Supplementary materials include 3 schemes, 4 figures, and 11 tables. Scheme S1: *N*,*N*-diethylamide derivatives (1)–(4) synthesis by Oxalyl Chloride, Scheme S2: Varying the Dihedral angle of C-C-N-C (α, β) on Aryl-CO bond and N-C bond, Scheme S3: Angles of Amide Structure, Figure S1: 2D-EXSY spectra of *N*,*N*-diethylamide derivatives (1),(2), Figure S2: The Potential Energy Surface Graph of Aryl-CO (a) and C-N (b) rotation on NCDEA, Figure S3: The Potential Energy Surface Graph of Aryl-CO (a) and C-N (b) rotation on PCDEA, Figure S4: Structure of ground state and transition states (QST3) of Aryl-CO bond and C-N bond in NCDEA and PCDEA, Table S1: Comparisons of Gibbs Free energy of ^1^H VT-NMR with those of scan coordinates calculated with the gaussian09 program, Table S2: The imaginary frequency on NCDEA and PCDEA, Table S3: Experimental and Theoretical Gibbs free energy of Aryl-CO bond in NCDEA, Table S4: Experimental and Theoretical Gibbs free energy of Aryl-CO bond in PCDEA, Table S5: Structure data of 2TS (diethyl-rotated conformers) of Aryl-CO bond in NCDEA and PCDEA, Table S6: Structure data of 2TS (diethyl-rotated conformers) of C-N bond in NCDEA, Table S7: Structure data of 2TS (diethyl-rotated conformers) of C-N bond in PCDEA, Table S8: Structure data of 2TS (diethyl-rotated conformers) of C-N bond in NCDEA and PCDEA, Table S9: Structure data of TS of the concerted Aryl-CO/C-N bond in NCDEA and PCDEA, Table S10: Structure data of TS of the concerted Aryl-CO/C-N bond of 2D PES in NCDEA and PCDEA, Table S11: Electron density of rotating bonds.
