# Supplementary material for: Electronic Effect on the Molecular Motion of Aromatic Amides: Combined Studies Using VT-NMR and Quantum Calculations
Source: Molecules. 2018 Sep 8;23(9):2294. doi: 10.3390/molecules23092294 (PMC6225462; doi:10.3390/molecules23092294)
Supplement: Supplementary file 1 [file molecules-23-02294-s001.pdf]

Supplementary Materials: The electronic effect on the molecular motion of aromatic amides: combined studies by VT-NMR and quantum calculations

Scheme S1. N,N-diethylamide derivatives (1)-(4) synthesis by Oxalyl Chloride

(a)

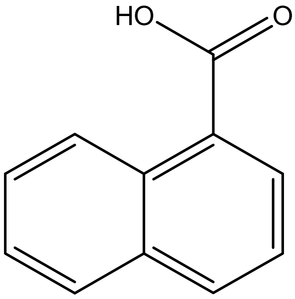

1-naphthoic acid (1)

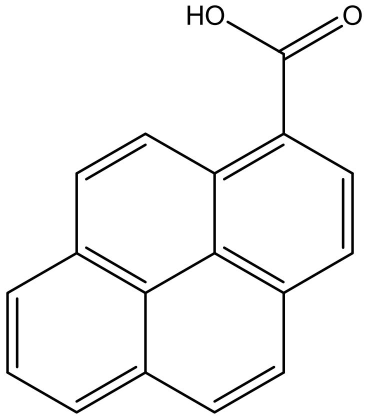

pyrene-1-carboxylic acid (2)

(b)

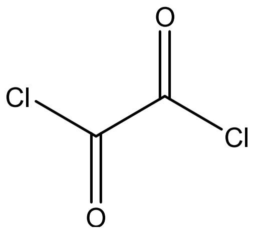

Oxalyl dichloride

(c)

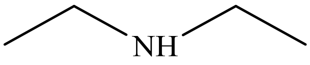

Diehtylamine

Scheme S2. Varying the Dihedral angle of C-C-N-C (α, β) on Aryl-CO bond and N-C bond.

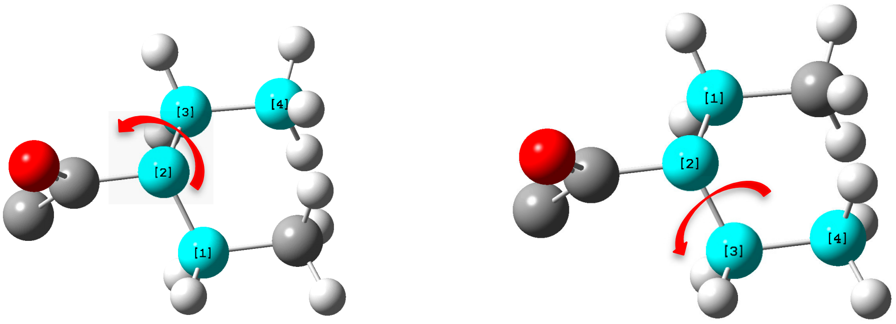

(a) α (b) β

Scheme S3. Angles of Amide Structure

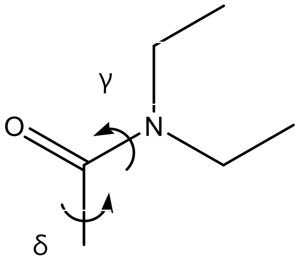

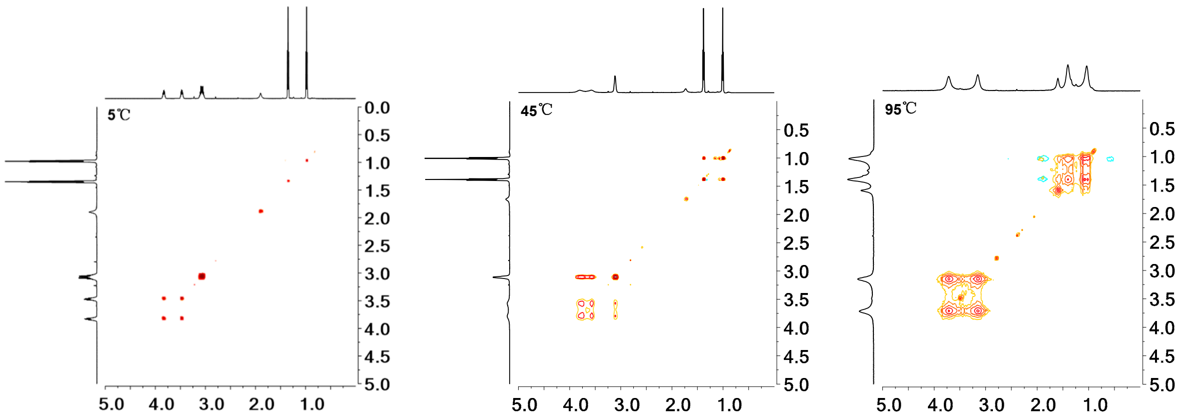

NCDEA

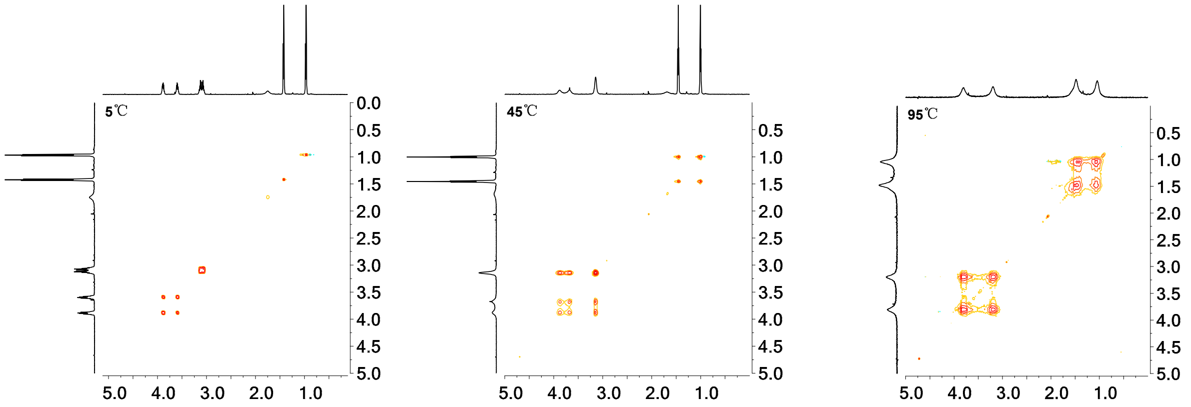

PCDEA

Figure S1. 2D-EXSY spectra of N,N-diethylamide derivateves (1),(2) (in unit of ppm in chemical shifts)

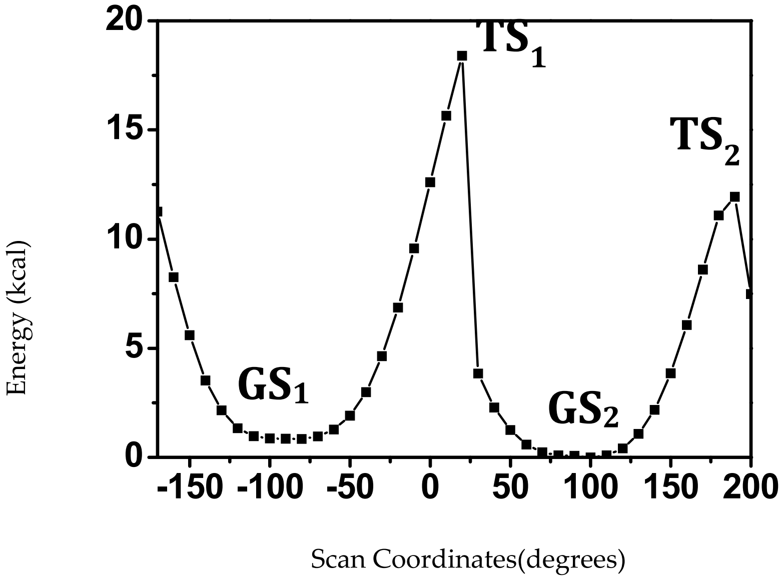

(a) Aryl-CO rotation

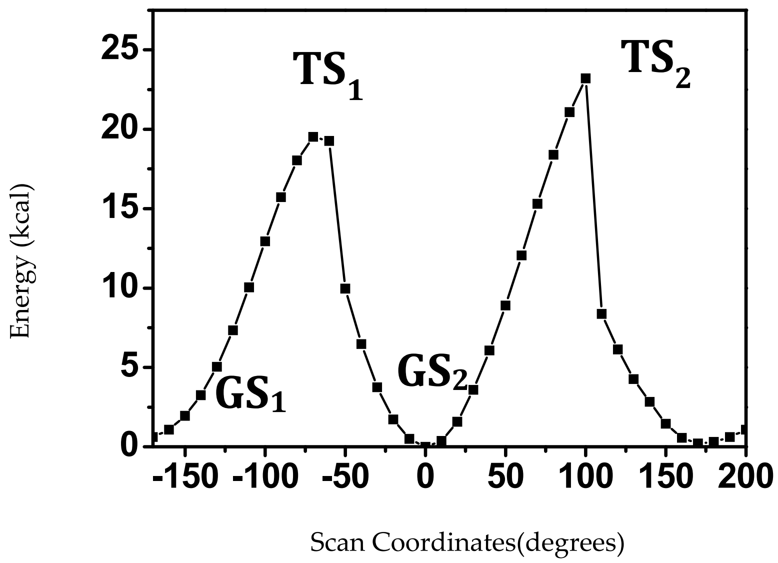

(b) C-N rotation

Figure S2. The Potential Energy Surface Graph of Aryl-CO (a) and C-N (b) rotation on NCDEA

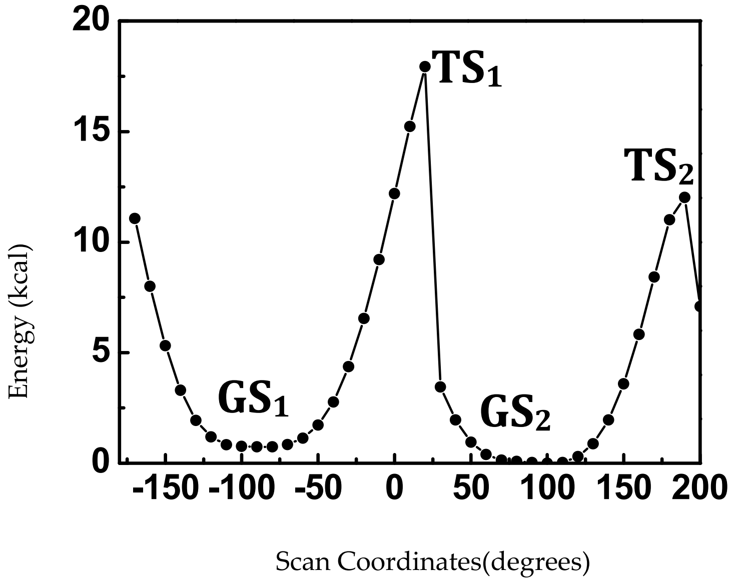

(a) Aryl-CO rotation

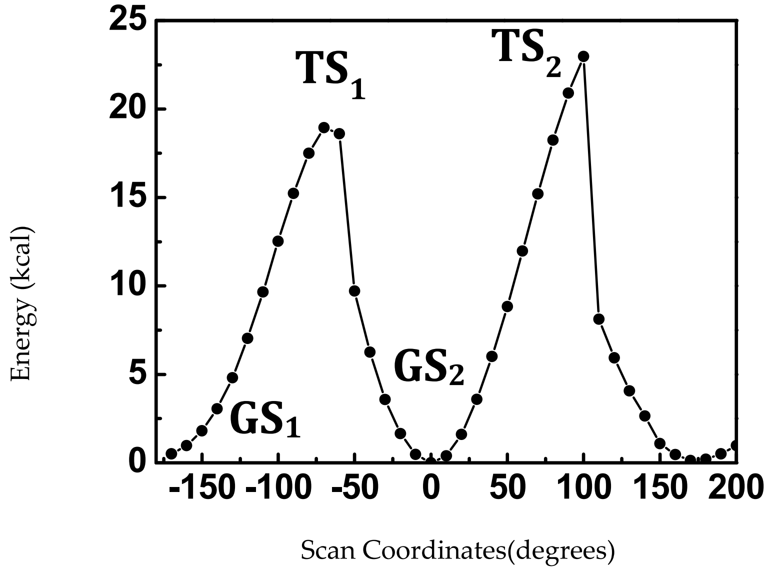

(b) C-N rotation

Figure S3. The Potential Energy Surface Graph of Aryl-CO (a) and C-N (b) rotation on PCDEA

GS1

α=107.34°

β=-67.96°

γ=1.99°

δ=94.12°

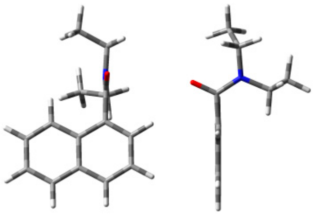

GS2

α=-88.41°

β=-80.16°

γ=-0.75°

δ=-74.53°

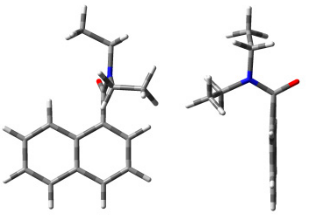

TS2 (QST3)

α=-86.77°

β=-93.77°

γ=-6.44°

δ=6.61°

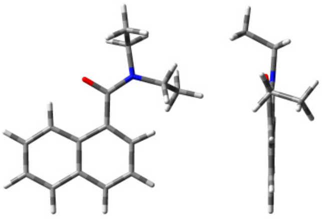

Aryl-CO bond

GS1

α=87.32˚

β=78.69˚

γ=-170.00˚

δ=-91.81˚

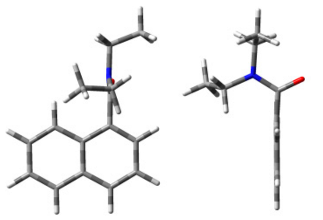

GS2

α=88.60˚

β=80.57˚

γ=0˚

δ=75.56˚

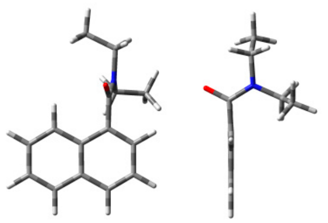

TS1 (QST3)

α=54.84˚

β=62.76˚

γ=-65.01˚

δ=-170.83˚

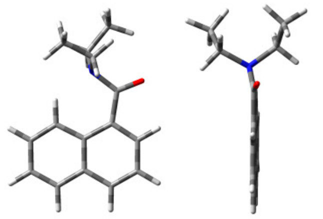

TS2 (QST3)

α=62.82˚

β=54.84˚

γ=64.99˚

δ=1.94˚

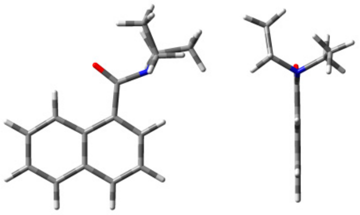

C-N bond

NCDEA

GS1

α=107.50˚

β=-68.32˚

γ=1.99˚

δ=93.88˚

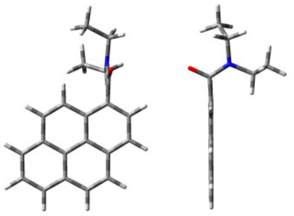

GS2

α=-89.00˚

β=-78.84˚

γ=-0.34˚

δ=-74.22˚

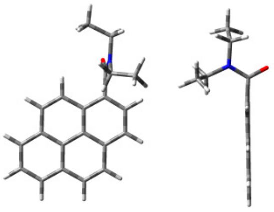

TS2 (QST3)

α=-86.75˚

β=-93.15˚

γ=-6.82˚

δ=6.58˚

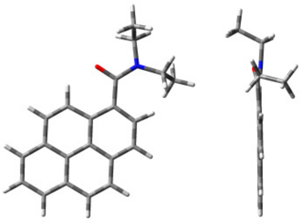

Aryl-CO bond

GS1

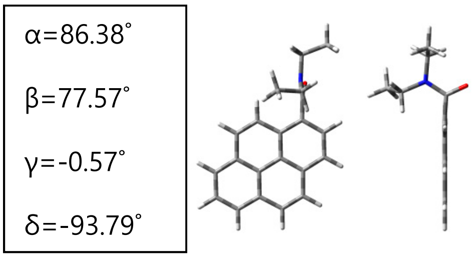

GS2

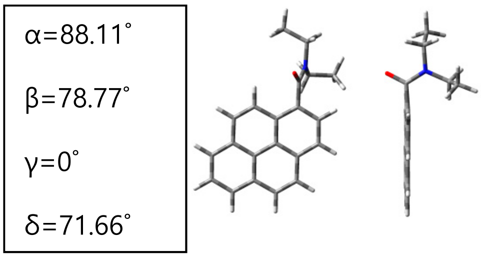

TS1 (QST3)

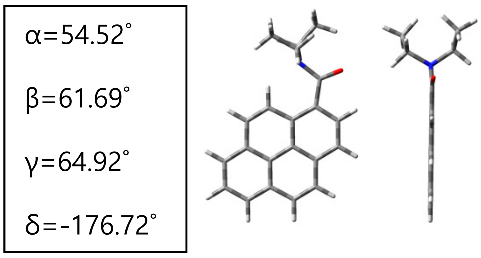

TS2 (QST3)

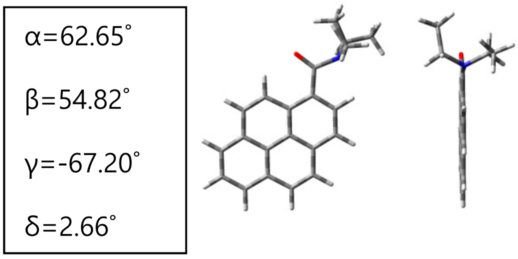

C-N bond

PCDEA

Figure S4. Structure of ground state and transition states (QST3) of Aryl-CO bond and C-N bond in NCDEA and PCDEA

Table

Table S1. Comparisons of Gibbs Free energy of Variable Temperature ^{1}H NMR with those of Scan cooridnates calculated with gaussian09 program

Compound	Bond	. Variable Temperature ^{1}H NMR $\Delta G^{\ddagger}/kcal\cdot mol^{-1}$	Gaussian09 (Scan coordinates*) $\Delta G^{\ddagger}/kcal\cdot mol^{-1}$
NCDEA	C-N / Aryl-CO	18.00	20.63/23.13
	Aryl-CO	15.40	20.10/13.96
PCDEA	C-N / Aryl-CO	17.65	19.55/23.13
	Aryl-CO	15.62	18.93/13.11

*Two Transition states are assumed in Scan coordinates

Table S2. The imaginary frequency on NCDEA and PCDEA

Compound	Aryl-CO bond rotation 1TS	Aryl-CO bond rotation 2TS (QST3)	The concerted Aryl-CO/C-N bond rotation 1TS (QST3)	The concerted Aryl-CO/C-N bond rotation 2TS (QST3)
NCDEA	None	-62.87	-78.23	-72.11
PCDEA	None	-60.53	-81.77	-73.34

*(in unit of cm^{-1})

Table S3. Experimental and Theoretical Gibbs free energy of Aryl-CO bond in NCDEA

2TS

Experimental Analysis (Variable Temperature ^{1}H NMR)		Theoretical Analysis (Gaussian09 program)
$\Delta G^{\ddagger}$/kcal·mol^{-1}	2TS	$\Delta G^{\ddagger}$/kcal·mol^{-1}
	Scan coordinates	13.96
	QST3 optimization	14.24
	Di-ethyl rotation 11	14.24
	Di-ethyl rotation 12	14.89
	Di-ethyl rotation 13	14.89
15.40	Di-ethyl rotation 21	15.19
	Di-ethyl rotation 22	Not Corresponding to Aryl-CO bond because of the number of frequency
	Di-ethyl rotation 23	Not Corresponding to Aryl-CO bond because of the number of frequency
	Di-ethyl rotation 31	15.19
	Di-ethyl rotation 32	Not Corresponding to Aryl-CO bond because of the number of frequency
	Di-ethyl rotation 33	Not Corresponding to Aryl-CO bond because of the number of frequency

*Di-ethyl rotation (1: -60° 2: 60° 3: 180°)

Table S4. Experimental and Theoretical Gibbs free energy of Aryl-CO bond in PCDEA

2TS

Experimental Analysis (Variable Temperature ^{1}H NMR)		Theoretical Analysis (Gaussian09 program)
$\Delta G^{\ddagger}$/kcal·mol^{-1}	2TS	$\Delta G^{\ddagger}$/kcal·mol^{-1}
15.62	Scan coordinates	13.11
	QST3 optimization	13.52
	Di-ethyl rotation 11	13.52
	Di-ethyl rotation 12	Not Corresponding to Aryl-CO bond because of the number of frequency
	Di-ethyl rotation 13	15.74
	Di-ethyl rotation 21	14.37
	Di-ethyl rotation 22	13.38
	Di-ethyl rotation 23	13.38
	Di-ethyl rotation 31	14.37
	Di-ethyl rotation 32	13.38
	Di-ethyl rotation 33	13.52

*Di-ethyl rotation (1: -60° 2: 60° 3: 180°)

Table S5. Structure data of 2TS (diethyl-rotated conformers) of Aryl-CO bond in NCDEA and PCDEA

(a) NCDEA

<table>
<tr><th></th><th>α</th><th>β</th><th>γ</th><th>δ</th></tr>
<tr><td>Di-ethyl rotation 11</td><td>-86.76°</td><td>-93.77°</td><td rowspan="4">-6.44°</td><td rowspan="4">6.61°</td></tr>
<tr><td>Di-ethyl rotation 12</td><td>-110.53°</td><td>-92.93°</td></tr>
<tr><td>Di-ethyl rotation 13</td><td>110.55°</td><td>-92.92°</td></tr>
<tr><td>Di-ethyl rotation 21</td><td>-85.48°</td><td>102.07°</td></tr>
<tr><td>Di-ethyl rotation 22</td><td colspan="4">Not Corresponding to Aryl-CO bond because of the number of frequency</td></tr>
<tr><td>Di-ethyl rotation 23</td><td colspan="4">Not Corresponding to Aryl-CO bond because of the number of frequency</td></tr>
<tr><td>Di-ethyl rotation 31</td><td>-85.51°</td><td>102.06°</td><td>-6.44°</td><td>6.61°</td></tr>
<tr><td>Di-ethyl rotation 32</td><td colspan="4">Not Corresponding to Aryl-CO bond because of the number of frequency</td></tr>
<tr><td>Di-ethyl rotation 33</td><td colspan="4">Not Corresponding to Aryl-CO bond because of the number of frequency</td></tr>
</table>

(b) PCDEA

	α	β	γ	δ
Di-ethyl rotation 11	-86.75°	-93.13°	-6.82°	6.58°
Di-ethyl rotation 12	Not Corresponding to Aryl-CO bond because of the number of frequency			
Di-ethyl rotation 13	111.62°	-91.95°		
Di-ethyl rotation 21	-85.23°	102.80°		
Di-ethyl rotation 22	96.94°	97.95°		
Di-ethyl rotation 23	96.95°	97.95°	-6.82°	6.58°
Di-ethyl rotation 31	-85.24°	102.79°		
Di-ethyl rotation 32	96.93°	97.96°		
Di-ethyl rotation 33	-86.75°	-93.13°		

Table S6. Experimental and Theoretical Gibbs free energy of C-N bond in NCDEA

Experimental Analysis (Variable Temperature ^{1}H NMR)		Theoretical Analysis (Gaussian09 program)
$\Delta G^{\ddagger}$/kcal·mol^{-1}	2TS	$\Delta G^{\ddagger}$/kcal·mol^{-1}
18.00	QST3 optimization	17.16
	Di-ethyl rotation 11	17.09
	Di-ethyl rotation 12	17.39
	Di-ethyl rotation 13	16.09
	Di-ethyl rotation 21	20.04
	Di-ethyl rotation 22	17.16
	Di-ethyl rotation 23	17.32
	Di-ethyl rotation 31	17.29
	Di-ethyl rotation 32	16.23
	Di-ethyl rotation 33	16.66

Table S7. Experimental and Theoretical Gibbs free energy of C-N bond in PCDEA

Experimental Analysis (Variable Temperature ^{1}H NMR)		Theoretical Analysis (Gaussian09 program)
$\Delta G^{\ddagger}$/kcal·mol^{-1}	2TS	$\Delta G^{\ddagger}$/kcal·mol^{-1}
17.65	QST3 optimization	17.04
	Di-ethyl rotation 11	17.26
	Di-ethyl rotation 12	17.40
	Di-ethyl rotation 13	15.97
	Di-ethyl rotation 21	19.92
	Di-ethyl rotation 22	17.04
	Di-ethyl rotation 23	17.01
	Di-ethyl rotation 31	17.08
	Di-ethyl rotation 32	16.27
	Di-ethyl rotation 33	16.50

Table S8. Structure data of 2TS (diethyl-rotated conformers) of C-N bond in NCDEA and PCDEA

(a) NCDEA

2TS	α	β	γ	δ
Di-ethyl rotation 11	-62.56	-55.44		
Di-ethyl rotation 12	-107.51	66.54		
Di-ethyl rotation 13	-74.00	166.48		
Di-ethyl rotation 21	60.67	-83.47		
Di-ethyl rotation 22	54.84	62.81	64.99	1.94
Di-ethyl rotation 23	58.32	162.98		
Di-ethyl rotation 31	-162.34	-58.61		
Di-ethyl rotation 32	-167.59	73.31		
Di-ethyl rotation 33	-167.43	167.37		

(b) PCDEA

2TS	α	β	γ	δ
Di-ethyl rotation 11	-63.11	-55.18		
Di-ethyl rotation 12	-112.10	67.43		
Di-ethyl rotation 13	-74.21	166.13		
Di-ethyl rotation 21	60.81	-83.58		
Di-ethyl rotation 22	54.81	62.67	-67.20	2.66
Di-ethyl rotation 23	58.61	162.94		
Di-ethyl rotation 31	-164.09	-58.92		
Di-ethyl rotation 32	-166.84	73.75		
Di-ethyl rotation 33	-167.40	167.75		

Table S9. Structure data of TS of the concerted Aryl-CO/C-N bond in NCDEA and PCDEA

Experimental Analysis (Variable Temperature ^1H NMR)	2D	Theoretical Analysis (Gaussian09 program)
$\Delta G^{\ddagger}$/kcal·mol^{-1}	TS	$\Delta G^{\ddagger}$/kcal·mol^{-1}
18.00	NCDEA	17.02
17.65	PCDEA	16.73

Table S10. Structure data of TS of the concerted Aryl-CO/C-N bond of 2D PES in NCDEA and PCDEA

TS	α	β	γ	δ
NCDEA	54.76	63.28	69.99	-1.81
PCDEA	53.11	62.92	69.99	6.21

Table S11. Electron density of rotating bond

			Electron density(∣e∣/Bohr^3)
NCDEA	aryl-CO	GS	0.2562
		2TS	0.2472
	C-N/aryl-CO	GS	0.3127
		2TS	0.2845
PCDEA	Aryl-CO	GS	0.2557
		2TS	0.2478
	C-N/aryl-CO	GS	0.3121
		2TS	0.2840
